# Forecasting the Fatigue Strength of DC01 Cold-Formed Angles Using the Anisotropic Barlat Model

**DOI:** 10.3390/ma15238436

**Published:** 2022-11-26

**Authors:** Mateusz Miksza, Łukasz Bohdal, Paweł Kałduński, Radosław Patyk, Leon Kukiełka

**Affiliations:** 1 Doctoral School, Koszalin University of Technology, Śniadeckich 2 Street, 75-900 Koszalin, Poland; 2 Department of Mechanical Engineering, Koszalin University of Technology, Racławicka 15-17 Street, 75-620 Koszalin, Poland

**Keywords:** bending, FEM analysis, Barlat’s anisotropy, springback, fatigue, strain life, total equivalent strain

## Abstract

The objective of this work is to present the numerical simulation of the air-bending process of DC01 steel. There are plenty of works concerned with assessing the springback phenomenon in the bending process also using anisotropic material models (Hill’s model is widely used). However, very few recent publications are concerned about the fatigue life assessment of bent products. As ensuring the proper fatigue resistance of products is vital for increasing safety and widening the service intervals there is certainly a need to perform investigations in this field. In this work, the air bending simulation of anisotropic DC01 steel with the usage of Barlat’s plastic anisotropy model was presented. Together with springback analysis and the equivalent plastic strain cumulation during incremental bending. Strain cumulation is believed to be an important factor in predicting fatigue life. It was shown that the strain development rate depends on the bending process parameters, especially from the bending line orientation to the sheet rolling direction.

## 1. Introduction

Sheet metal bending is widely used in numerous branches of industry, starting from production of small decorative elements in furniture production through the automotive and aviation industry. It is not a separated case when bent elements perform a responsible design function acting as holders, supports, or hangers for equipment. Thus, quality requirements in relation to them are really high. Mainly in terms of proper dimensional and shape accuracy, but also fatigue life expectancy is an important factor as it translates straightforward to operational safety and widening the service intervals.

Although the bending process is well-known and widely used it is still the subject of thorough scientific investigation. The main areas of concern are the prediction and suppression of the springback phenomenon and the technical issues connected to the shaping of anisotropic materials. In work [1], for the springback prediction of the hat-shaped part made of advanced high-strength dual-phase steel HCT600X+Z numerical simulation was used. Numerical predictions were performed with the use of various combinations of material models to try to improve the prediction results. The finite element analysis was conducted to investigate the impact of used yield functions and hardening models on the springback prediction accuracy in numerical predictions. Two types of tool designs were used and their impact on the springback was evaluated.

Lawanwong et al. [2] conducted a “double-action bending” process to eliminate the springback of advanced high-strength steel. In order to analyze the influence of the process parameters on its course and product quality, they used FE analysis. In study [3], the bendability of the high-strength aluminum alloy sheets with two different thicknesses was experimentally evaluated by the V-die air bending test along with the punch radius and the material direction. The numerical simulation procedure was attempted to comprehend the characteristics of the V-die air bending method. In work [4] the failure phenomena and underlying damage mechanisms in small curvature bending of AHSS were analyzed. An optical strain measurement technique was presented to identify the failure stages, critical strains, and bending angles. In study [5], mini V-bending testing was conducted on 316L stainless steel specimens fabricated by selective laser melting (SLM). The evolution of the microstructure in the bending zone was analyzed. The microstructure analysis showed that raw SLM samples contain pores in the surface and subsurface regions, which influence deformation and crack behavior during the V-bending process. FE simulation was performed to understand the macroscopic deformation in the bend zone. It was found that the nucleation and propagation of cracks during the bending process are influenced by high equivalent states of plastic deformation and triaxial stresses in the regions near the pores on the surface and subsurface of the material.

Sheet metal anisotropy is a derivative of the material production process, where the sheet metal rolling process elongates the material grains in the rolling direction [6]. This phenomenon might be limited by multiple rolling passes, but it is connected with a great increasement in manufacturing price and reserved for premium quality materials which are often discarded in production due to the high material price.

It was proven by Bakhshi-Jooybari et al. [7], Baseri et al. [8], and Trzepieciński and Lemu [9,10] that the angle between bending line and sheet rolling direction—*θ*, influences the springback phenomenon. Mentioned authors say in unison that the most favorable case is when the bending line overlaps with the rolling direction (*θ* = 0°), but this is not always achievable due to technological and design reasons.

Other factors influencing the springback phenomenon are the material strength properties such as Young’s modulus and yield strength [11], material thickness, and bending radius [7,8,12].

Although the bending process is widely used, there are no significant correlations between process parameters and fatigue life of bent products shown in the available literature. Most of the works concerned about fatigue cracking focus on the investigation of the material structure, showing that the grain boundary area is the crack initiation site [13,14]. Although Hou et al. [14] connected the plastic deformations with the crack growth rate, their work focuses on rolled samples and they do not take the plastic working such as bending into consideration. Takahashi et al. [15] and Suárez Fernández et al. [16] proved that fatigue crack propagates along the grain boundaries and shows the anisotropic direction of propagation spreading more easily on the material surface than in thickness.

Mattei et al. [17] and Muhammad et al. [18] took up the issue of material bendability, taking into account the material structure. It was shown that crack initiation due to the overbend started at the surface grain boundary area. This is similar to the fatigue crack initiation area mentioned in [13,14,15,16]. After summarizing the sheet metal anisotropy mentioned in [6], dependence of spingback phenomenon of the angle between bending line and the rolling direction (*θ*) shown in [7,8,9,10], fatigue crack initiation and propagation conditions described in [13,14,15,16] with the findings presented in [17,18] one can draw a clear conclusion that the fatigue life expectancy is connected with the bending process parameters and sheet metal anisotropy. Due to the fact that this issue has not been previously investigated, there is a need for research in this field.

According to the ASM International Elements of Metallurgy and Engineering Alloys Handbook, fatigue damage is the most common reason for mechanical service failures. Approximately 90% of failure cases are a consequence of the effect of fatigue [19]. Hence, it is important to obtain knowledge of how bending process parameters influence the proposed fatigue life of the manufactured part. This may lead to the determination of guidelines set for the proper design of bending processes in terms of achieving the longest possible fatigue life and service intervals. The aim of the presented article is to develop regression functions of cumulative strain development in case of multiple bending of parts manufactured by air bending with various process parameters, which allow for the prediction of workpiece fatigue life.

**Figure 1:**
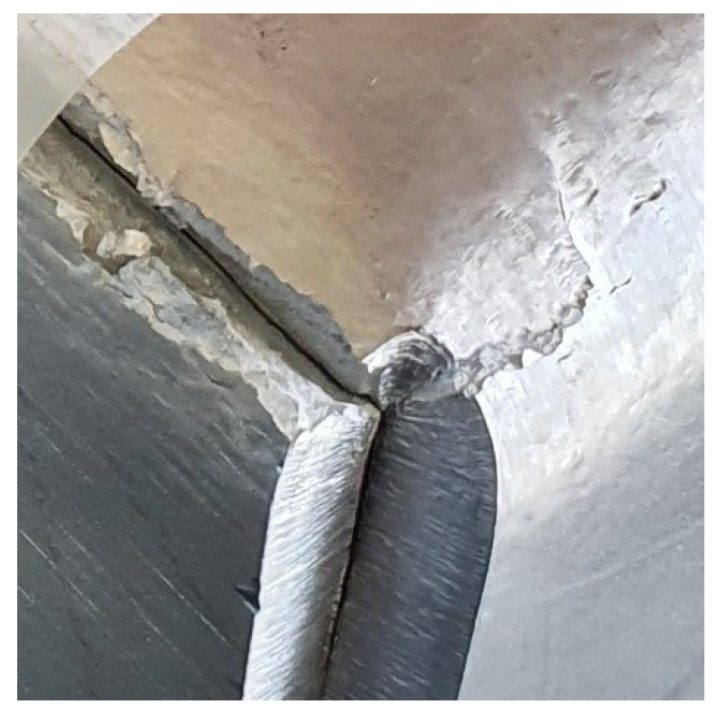
Edge cracking along the bending line caused by the wrong (too low) bending radius.

**Figure 2:**
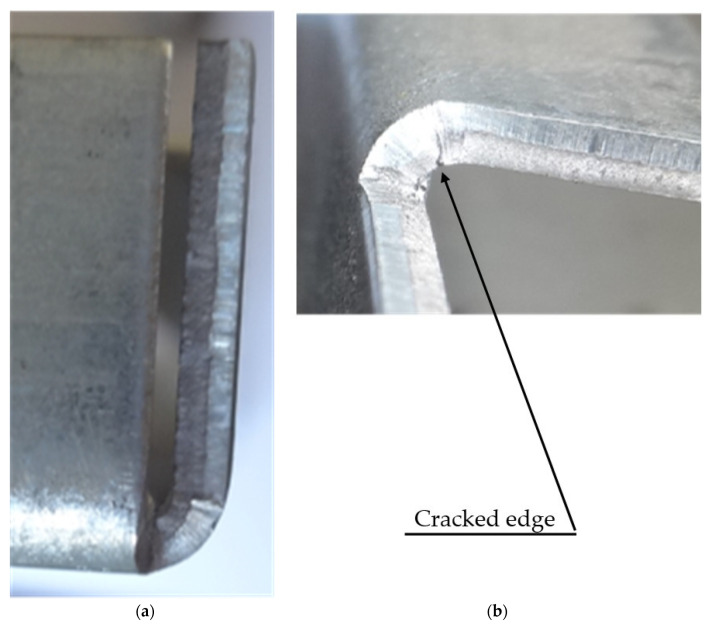
Sheet anisotropy influence on the manufactured part quality. Bending along the sheet rolling direction (**a**) and transverse to it (**b**).

Bending processes are sensitive to the choice of the proper parameters. Wrong parameters may lead to significant failures of manufactured parts. One of the most common cases is a crack along the bending line caused by the too-low bending radius which was observed during the authors’ research and is shown in Figure 1, while the influence of sheet metal anisotropy can be observed in Figure 2.

The presented part was manufactured by bending along the sheet rolling direction and perpendicular to it using the same bending parameters. As can be observed on Figure 2a, the edge bent along the rolling direction is failure free, but the one bent in the transverse direction is cracked, which is shown on Figure 2b.

## 2. Materials and Methods

Actual tendency in industrial production is to minimize the preparatory time. In case of the sheet metal bending this task is realized by using the air bending process, which allows for achieving multiple bending angles on various material thicknesses without the change of bending tools.

**Figure 3:**
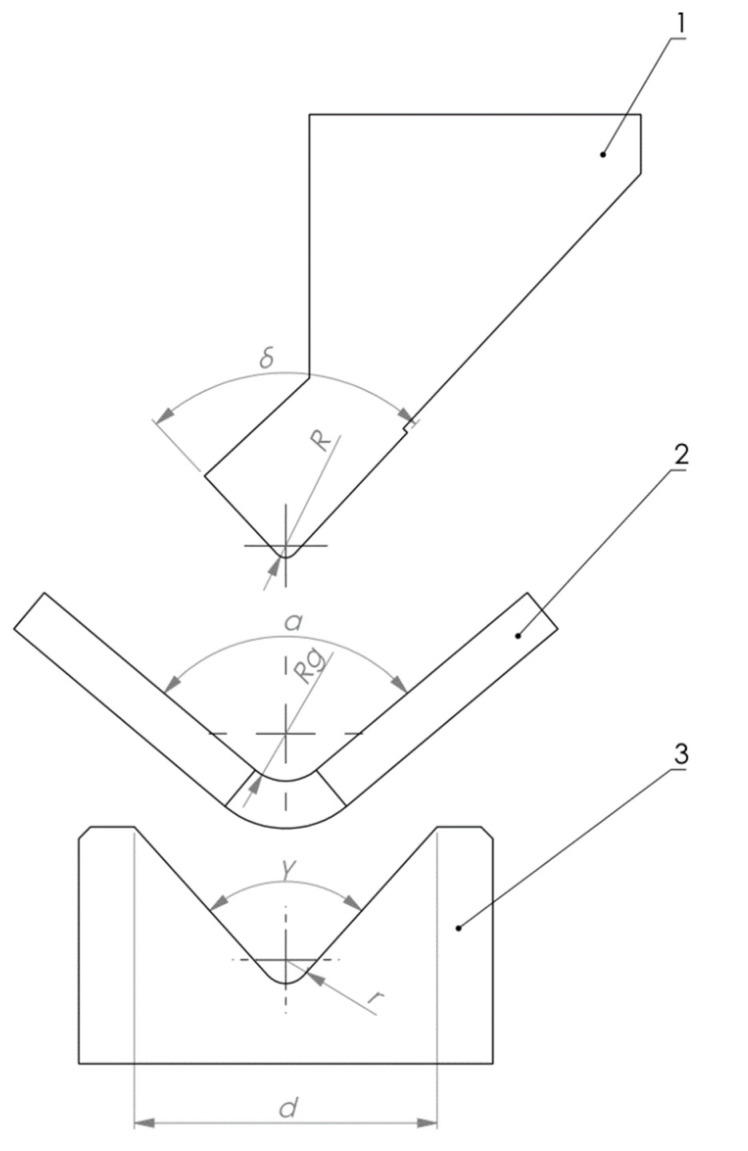
Schematic representation of air bending process. 1—punch, 2—sheet metal, 3—die. Letter and numerical markings was described in the text.

The scheme of air bending process is shown in Figure 3. Material (numerical marking—2) is pressed into the die (numerical marking—3) using the punch (numerical marking—1). In the air bending it is not required for the material to reach the die bottom. This is a very efficient and time-saving technique. The bending radius–*Rg* is determined by the combination of die width—*d*, die angle—*γ,* and punch tip radius—*R*. While the bending angle—*α* is determined by the punch translation in vertical axis. Parameters such as die bottom radius—*r* and punch angle—*δ* do not show a great influence on the air bending process.

**Figure 4:**
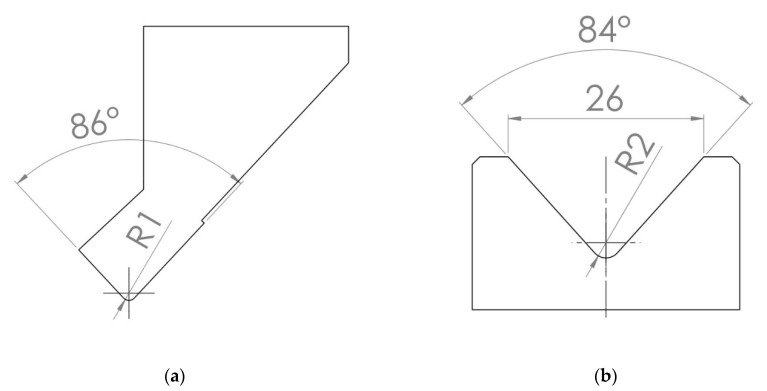
The geometry of the TPR256.86.R1-A punch (**a**) and the TMR100.24.84 die (**b**) recreated using the manufacturer’s data.

For the case of air bending process simulation, the real industrial tool geometry was obtained courtesy of local production facility. As technical survey showed, sheet metal with a thickness of—*x* ≤ 4 mm is shaped with punch with tip radius—*R* = 1 mm. In this specific case, the Rolleri S.p.A.^®^ (located in Vigolzone, Italy) TPR256.86.R1-A punch, which is shown in Figure 4a, and TMR100.24.84 die, which is shown in Figure 4b were used. The geometry of tools is provided by the manufacturer on his website in dxf file format [20,21].

Thanks to the provided data, the tools were recreated in Dassault Systèmes SOLIDWORKS Corp. (located in Waltham, MA, USA) Solidworks^®^ software and then saved in neutral Parasolid (*.x_t) file format. This allowed for direct implementation in Ansys Inc. – Livermore Software Technology Corporation (located in Livermore, CA, USA) Ansys^®^ LS-Dyna™ environment. After importing the geometry, it was discretized using the constant stress solid elements with six points of integration. In case of bending the 4 mm thick sheet metal, the model of the process consists of 250236 finite elements. Authors performed analyses for various element types and sizes, which were followed by mesh convergence analysis. This study was performed using two methods—comparison of element and nodal results and minimizing differences between them and by analysis of dissipation energy and its minimalization. The final finite element mesh used in simulation is the outcome of described procedure.

Tools were considered as rigid bodies so material 020-Rigid was declared with parameters for structural steel (*ρ* = 7800 kg/m^3^; *E* = 210 GPa; *ν* = 0.33).

The shaped blank is manufactured from cold rolled, low carbon steel for plastic working (DC01) manufactured according to the PN-EN 10130 standard [22]. Material’s chemical composition is shown in Table 1.

As mentioned in [6] most sheet metal materials are anisotropic, which has great influence on their shaping process. Very efficient yield function for simulating the plastic working of anisotropic material was proposed by Frédéric Barlat et al. [23] in 1991. Barlat’s six-parameter function has been proven to be very useful for modeling plastic strains of anisotropic materials [23,24]. It contains six weight parameters describing the anisotropy (*a*, *b*, *c*, *f*, *g*, *h*) and takes the form as in Equation (1).

(1) $$\Phi ={\left(3I_{2}\right)}^{m/2}\left\{{\left[2cos\left(\frac{2\theta +\pi }{6}\right)\right]}^{m}+{\left[2cos\left(\frac{2\theta -3\pi }{6}\right)\right]}^{m}+{\left[-2cos\left(\frac{2\theta +5\pi }{6}\right)\right]}^{m}\right\}=2{\bar{\sigma }}^{m},$$

where:

(2) $$\theta =arccos\left(\frac{I_{3}}{I_{2}^{3/2}}\right),$$

and:

(3) $$I_{2}=\frac{{\left\{fF\right\}}^{2}+{\left\{gG\right\}}^{2}+{\left\{hH\right\}}^{2}}{3}+\frac{{\left(aA-cC\right)}^{2}+{\left(cC-bB\right)}^{2}+{\left(bB-aA\right)}^{2}}{54}\;$$

(4) $$\begin{array}{cc} I_{3}= & \frac{\left(cC-bB\right)\left(aA-cC\right)\left(bB-aA\right)}{54}+fghFGH\\ & -\frac{\left(cC-bB\right){\left\{fF\right\}}^{2}+\left(aA-cC\right){\left\{gG\right\}}^{2}+\left(bB-aA\right){\left\{hH\right\}}^{2}}{6}.\; \end{array}$$

The parameters set in Barlat’s plastic anisotropy model in Ansys Inc.–Livermore Software Technology Corporation (located in Livermore, CA, USA) LS-Dyna are shown in Table 2. Those parameters are calculated experimentally, and it has been proven they have a significant effect on the simulation accuracy. In this case, parameters were obtained based on the previous works of the authors [25].

**Figure 5:**
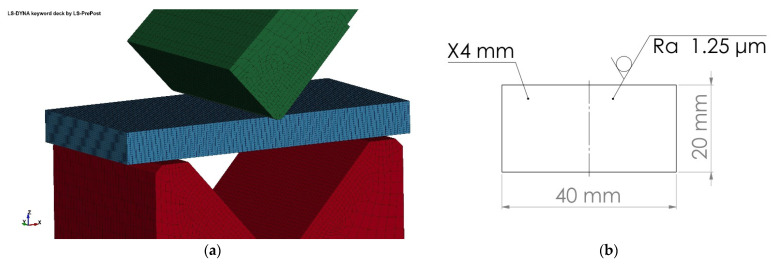
Discretized numerical model prepared in the LS-Dyna software (**a**) and dimensions of the undeformed specimen used in simulation (**b**).

Model prepared for simulation is presented in Figure 5a and dimensions of undeformed specimen are presented in Figure 5b. Contact between bodies was set using the AUTOMATIC_SINGLE_SURFACE method with static coefficient of friction—*μ_s_* = 0.1 and dynamic coefficient of friction *μ_d_* = 0.01. Friction coefficient values were determined experimentally using the materials testing machine ZwickRoell Z400E manufactured by Zwick Roell Group (located in Ulm, Germany).

**Figure 6:**
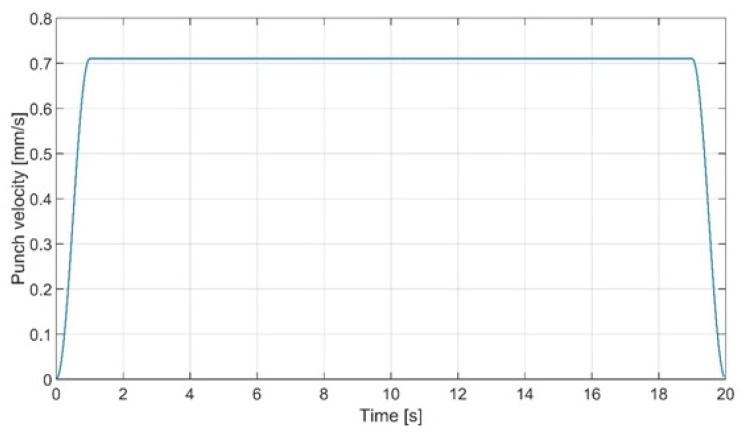
Punch displacement velocity curve.

Translational displacement of punch along the Z axis was declared using the velocity curve as shown in Figure 6. The maximal displacement of punch equals 13.5 mm, but in order to achieve the given values of bending angle the simulation was paused in the time steps corresponding to them and the deformed geometry with strain and stress history was exported as the input file for the subsequent analyses.

There were eight cases considered. Input parameters were: material thickness—*x* (*x* = 3 mm, *x* = 4 mm), bending angle—*α* (*α* = 90°, *α* = 115°), and angle between bending line and sheet rolling direction—*θ* (*θ* = 0°, *θ* = 90°). Study was performed using the five-level rotatable experiment plan and its range was adjusted to achieve desired results. This task required following steps to be conducted:

1. Determination of variability range of the studied parameters.
2. Choice of the class of the mathematical model.
3. Coding the analyzed parameters.
4. Gathering the experiment results.
5. Elimination of results with gross error.
6. Calculating the inter-row variance and standard deviation.
7. Checking the homogeneity of variance.
8. Calculating the coefficients of regression function.
9. Statistical analysis of the regression function.
10. Examination of the significance level of the correlation coefficient.
11. Checking the adequacy of the mathematical model.
12. Decoding the regression function.

After obtaining the deformed geometry of each case, the springback analysis was performed using the implicit solver. Then, the geometry with strain and stress history was once again used as input file for analysis of multiple bending.

**Figure 7:**
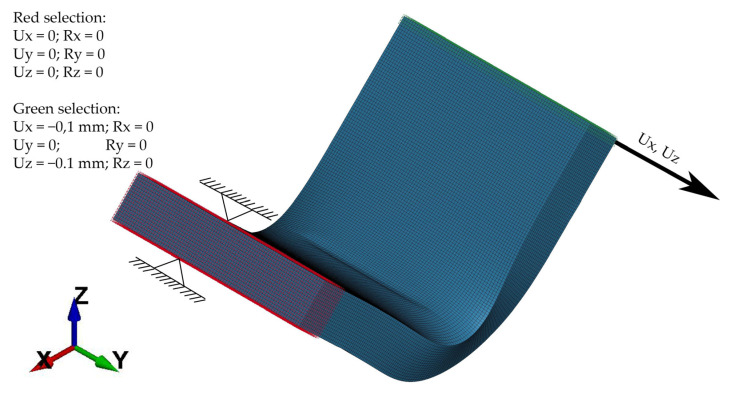
Specimen load scheme.

**Figure 8:**
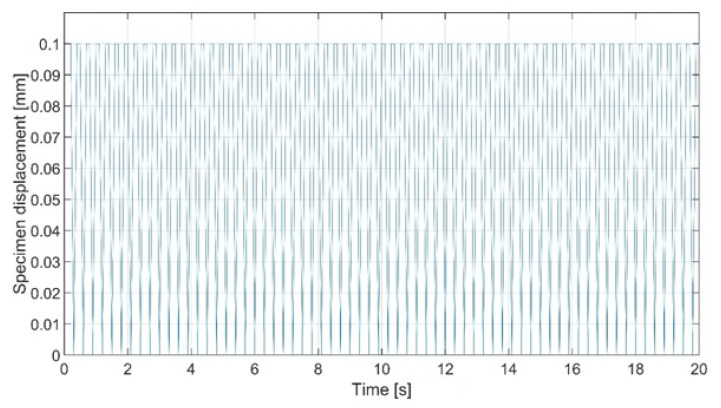
Specimen load curve.

In this case, the specimen was a subject of multiple unilateral loads which were applied perpendicularly to its surface, as shown in Figure 7. In each iteration, the displacement of 0.1 mm was applied. Load curve is shown in Figure 8.

## 3. Results

Following section provides the brief overview of obtained results with preliminary interpretation, which will be developed in detail in discussion section. The section is divided into subsections, which concerns each aspect of conducted analyses. In the first place, the simulation validation process is described. Then, the distribution of stresses and strains in the analyzed specimens is discussed. Subsequently, the results of the springback analysis are presented, and finally, the results of the simulation of the fatigue strength in the form of the plastic strain accumulation analysis in the finite element are presented.

### 3.1. Simulation Validation

**Figure 9:**
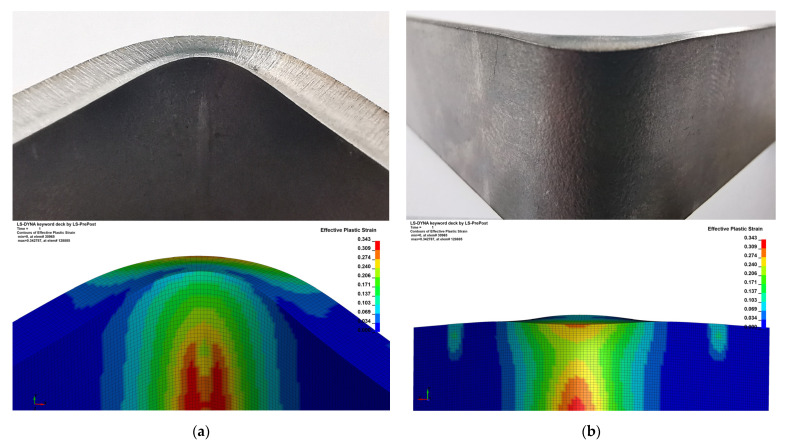
Qualitative comparison of plastic deformation in bending zone. (**a**)—punch side (**b**)—die side.

To validate the simulation results, the specimens of 3 mm thick DC01 steel were prepared using the industrial CNC brake press and mentioned tools. Then, obtained results of plastic deformation and springback coefficient were compared in qualitative and quantitative way. As shown in Figure 9a, a high level of correlation was obtained in terms of plastic deformations.

For the further validation, the analysis of material thickness in bending zone was conducted. After measuring the thickness variation between the bending zone and undeformed specimen, the authors discovered that it is about 1% of initial thickness. Therefore, this analysis is not the subject of simulation validation. Furthermore, those observations are in line with Marciniak et al. [26], who claims that thickness reduction in sheet metal bending process is negligible.

**Figure 10:**
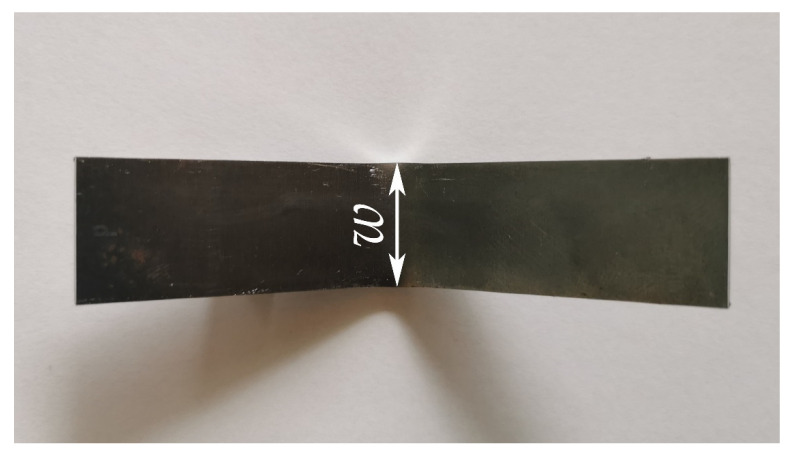
Illustration of width change measurement in bending zone, *w*—specimen width.

Additionally, the change in specimen width in the bending zone, which is marked as *w* in Figure 10 was assessed, results are shown in Table 3. The measurement of specimens was performed using a digital micrometer with 0.001 mm resolution. Thus, results from the simulation were also rounded to three decimal places.

The results of the springback comparison are shown in Table 4. Angle measurements were performed using a Vernier protractor with a resolution of 5′ and substituted into Equation (5).

The results presented above show the simulation can be considered valid. The maximal relative error value is 1.77% and other values do not exceed 1% which fits into the 95% confidence bounds. Although it has to be pointed out that the results from the simulation were overpredicted and the width change in the experiment was not as high as in the numerical solution. A possible explanation is the presence of heat affected zone near the edges of specimens used in the experiment, as they were cut using a laser cutter. The presence of this zone is neglected in the simulation. It is believed that edge hardness increasement due to the heat influence reduced plastic deformation.

Unlike the case of plastic deformation, the numerical results of springback coefficient value showed the underpredicted value. Although differences are noticeable, the relative error value fits between the 95% confidence bounds. It could be said, with high level of certainty, that those differences are induced by Young’s modulus value used in the simulation, where the default value for structural steel was used and the real value of specimens was not determined.

In the given case, where the strain numerical analysis has been performed, the usage of a strain gauge is considered to be substantial as a simulation validation instrument. Hence, the initial strain analysis was conducted in the Trilion Quality Systems (located in King of Prussia, PN, USA) ARAMIS strain analysis system, which gave satisfactory results. This opens future work possibilities as the strain measurement will be the objective of further publications.

### 3.2. Stress Analysis

**Figure 11:**
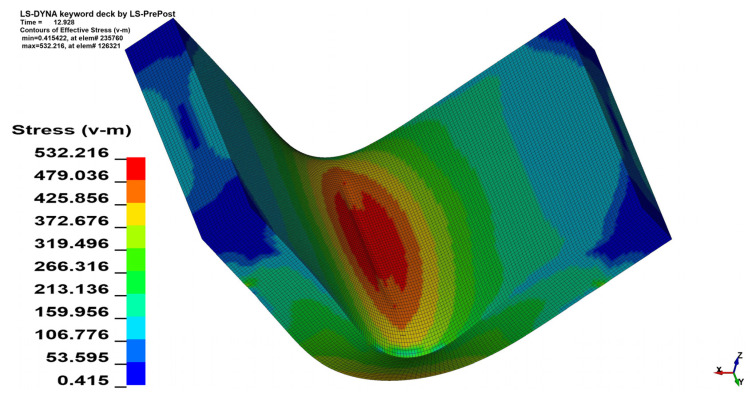
HMH equivalent stress distribution map for *x* = 4 mm, *α* = 90° and *θ* = 0°.

**Figure 12:**
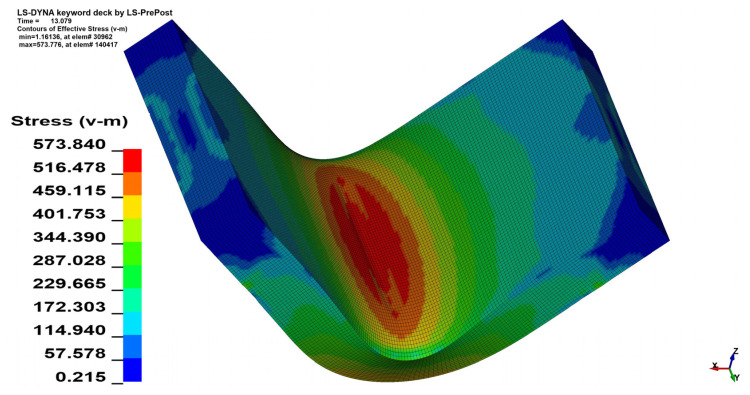
HMH equivalent stress distribution map for *x* = 4 mm, *α* = 90° and *θ* = 90°.

The stress analysis was performed using the Huber–von Mises–Hencky equivalent stress hypothesis (HMH) [27,28,29]. As can be seen in Figure 11 and Figure 12 the change in the *θ* angle from 0° to 90° influences the stress distribution. In the case of bending the 4 mm thick material on the angle *α* = 90° increment of the maximal registered stress value equals 41.6 MPa when *θ* = 90°, which is 7.82% of the initial stress value. Changes in maximal stress value in analyzed cases are shown in Table 5 (for *x* = 4 mm) and Table 6 (for *x* = 3 mm).

**Figure 13:**
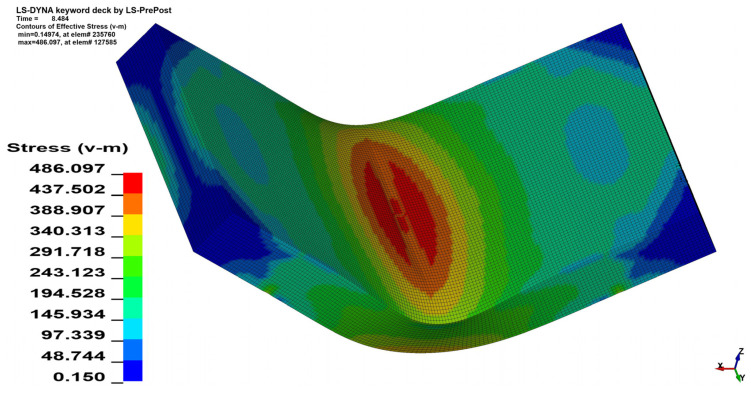
HMH equivalent stress distribution map for *x* = 4 mm, *α* = 115° and *θ* = 0°.

**Figure 14:**
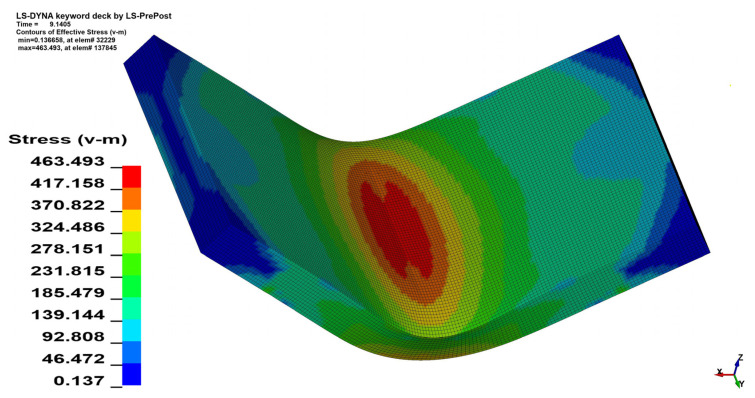
HMH equivalent stress distribution map for *x* = 3 mm, *α* = 115° and *θ* = 0°.

Reducing the material thickness also has an influence on the maximal stress value. In the case when *α* = 115° and *θ* = 0° reducing the plate thickness from *x* = 4 mm to *x* = 3 mm allowed for maximal stress reduction value by 22.6 MPa which stands for a 4.65% decrease. A comparison of stress maps for the given case can be seen in Figure 13 and Figure 14.

As can be seen in Table 5 and Table 6 another factor influencing the stress value and distribution is the bending angle—*α*. In general, lowering the angle increases the stress value, which can be observed in all analyzed cases. The percentage of change depends on the value of the *θ* angle and material thickness—*x*. The most significant change (8.66%) was observed for *θ* = 0° and *x* = 4 mm. While *θ* = 90° and *x* = 3 mm change in bending angle resulted in a 6.88% change in maximal stress value.

### 3.3. Plastic Strain Analysis

**Figure 15:**
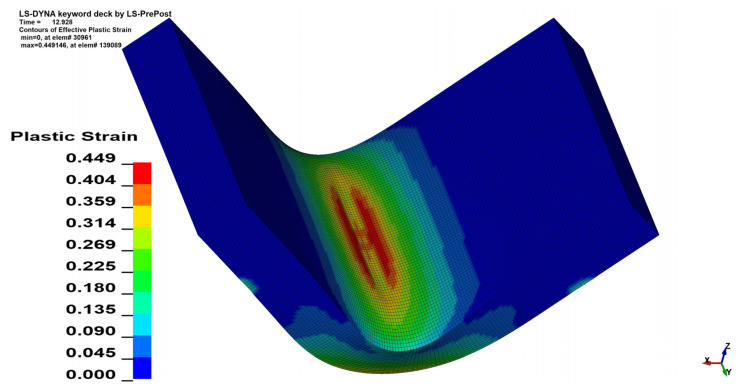
Equivalent plastic strain distribution map for *x* = 4 mm, *α* = 90° and *θ* = 0°.

**Figure 16:**
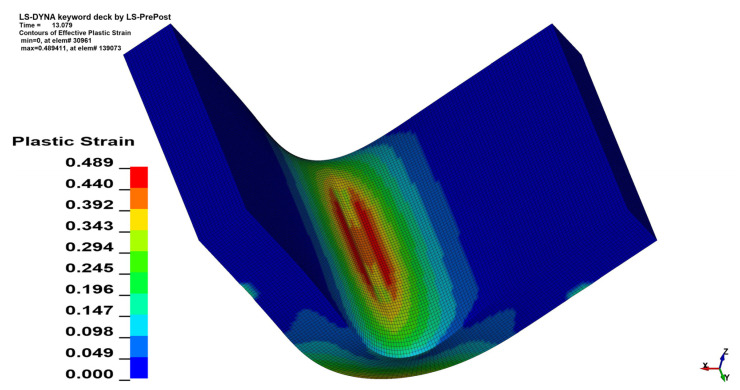
Equivalent plastic strain distribution map for *x* = 4 mm, *α* = 90° and *θ* = 90°.

Qualitative analysis of strain maps showed that change in *θ* value does not have a significant influence on strain distribution, which is shown in Figure 15 and Figure 16, but quantitative analysis showed the differences. As was shown in the stress analysis, bending perpendicularly to the sheet rolling direction (*θ* = 90°) increases the strain value similarly to the stress value increment. In the case of *x* = 4 mm, *α* = 90° the change in maximal equivalent strain equals—Δ*ε* = 0.0403 mm/mm, which corresponds to 8.97% of the initial value. The aggregate results of the analyzed cases are shown in Table 7 (*x* = 4 mm) and Table 8 (*x* = 3 mm).

As could be expected, reducing the material thickness has a similar effect on stress and strain values. In the case of *α* = 115° and *θ* = 0° the percentage reduction of plastic strain value between *x* = 4 mm and *x* = 3 mm equals 19.66% (0.0643 mm/mm).

### 3.4. Springback Coefficient Analysis

Springback coefficient—*K* is a dimensionless value which describes the difference between the final bending angle (after unloading)—*β* and the one achieved under the load—*α*. It is described by the Equation (5):

(5) $$K=\frac{\beta }{\alpha }$$

The value of the *K* coefficient was calculated using the formula (5) and deformed geometry measurements performed in LS-Dyna. The results are shown in Table 9.

It can be observed that all analyzed factors have an influence on the *K* coefficient value. Reducing the material thickness, bending angle and the angle between the bending line and rolling direction has a beneficial effect on reducing the scale of the springback phenomenon. While, reducing all of those values is almost impossible at one time due to technological, design, and economic reasons it is necessary to use optimization techniques for the proper design of the bending process.

### 3.5. Fatigue Analysis

**Figure 17:**
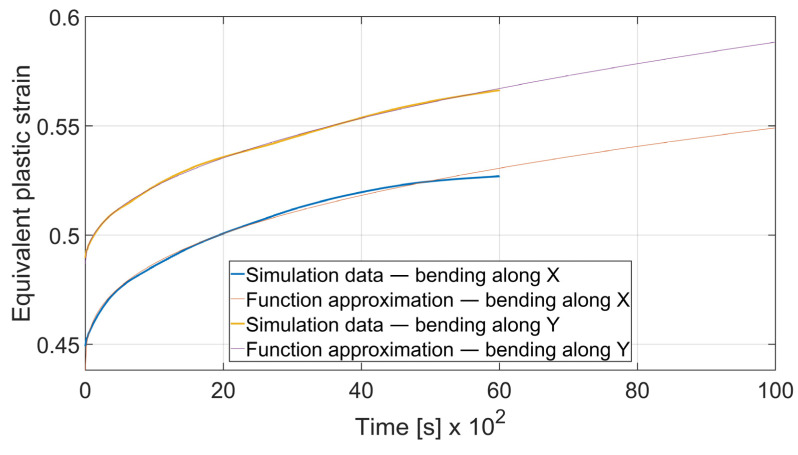
Equivalent plastic strain cumulation curves for *x* = 4 mm and *α* = 90°.

**Figure 18:**
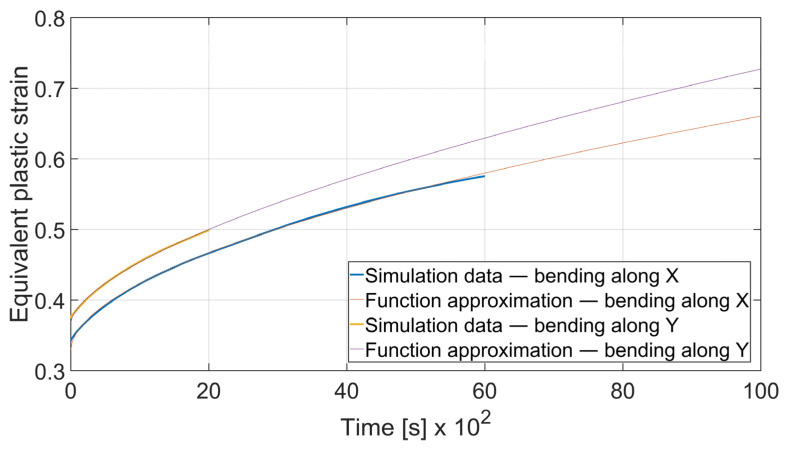
Equivalent plastic strain cumulation curves for *x* = 4 mm and *α* = 115°.

**Figure 19:**
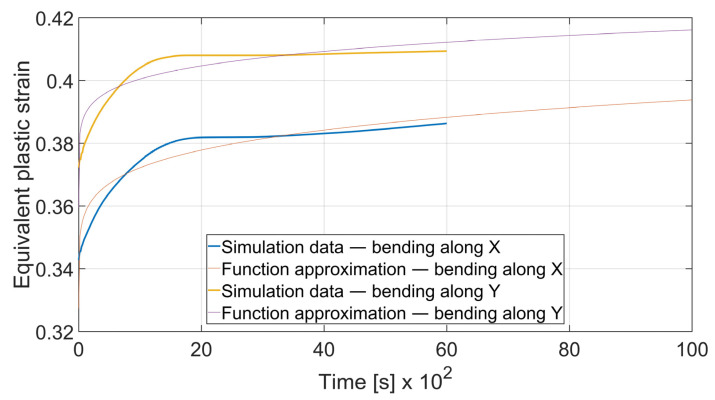
Equivalent plastic strain cumulation curves for *x* = 3 mm and *α* = 90°.

**Figure 20:**
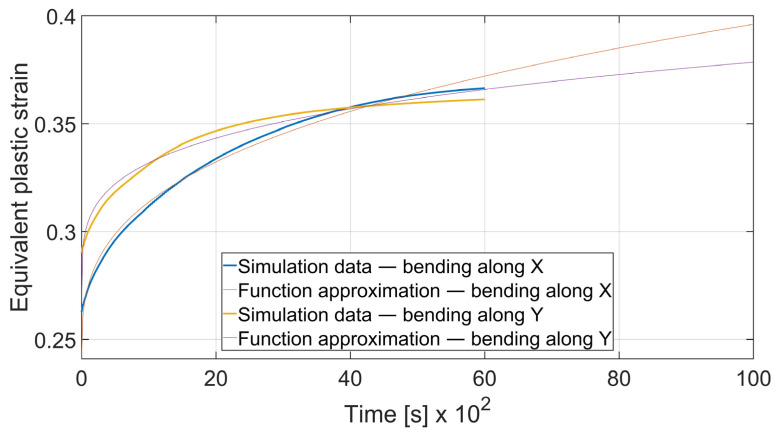
Equivalent plastic strain cumulation curves for *x* = 3 mm and *α* = 115°.

**Figure 21:**
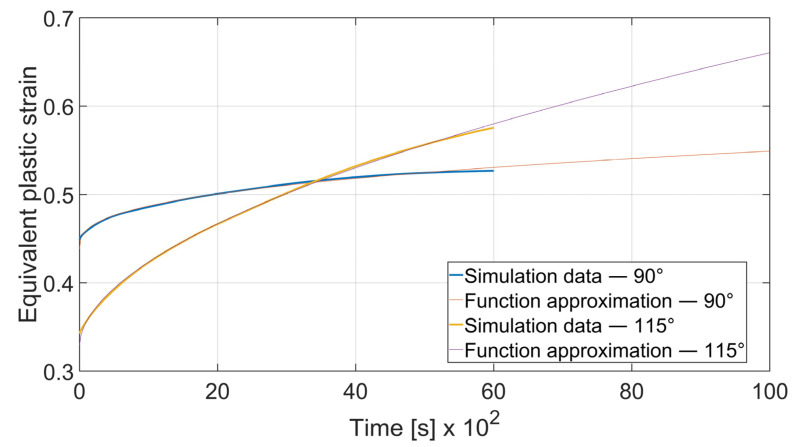
Equivalent plastic strain cumulation rate comparison for *x* = 4 mm and *θ* = 0°.

**Figure 22:**
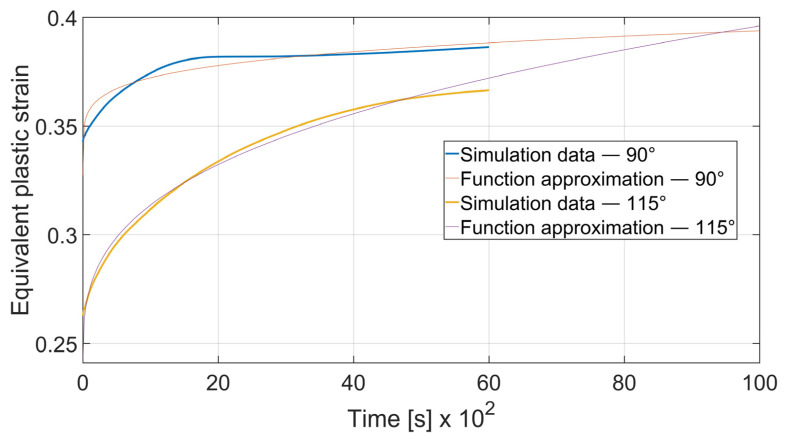
Equivalent plastic strain cumulation rate comparison for *x* = 3 mm and *θ* = 0°.

**Figure 23:**
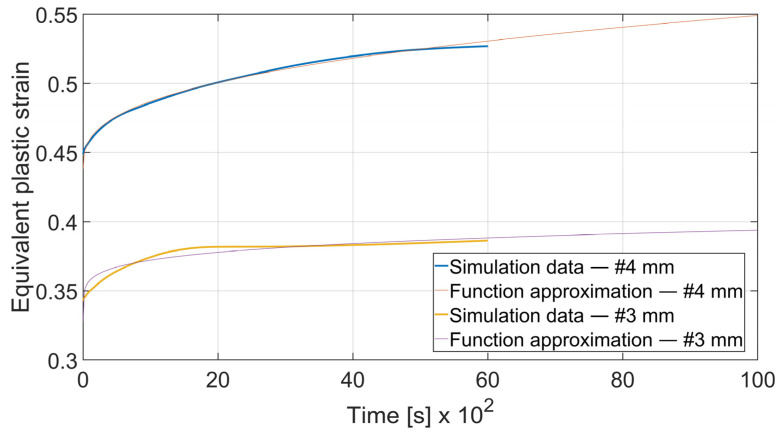
Equivalent plastic strain cumulation rate comparison for *α* = 90° and *θ* = 0°.

**Figure 24:**
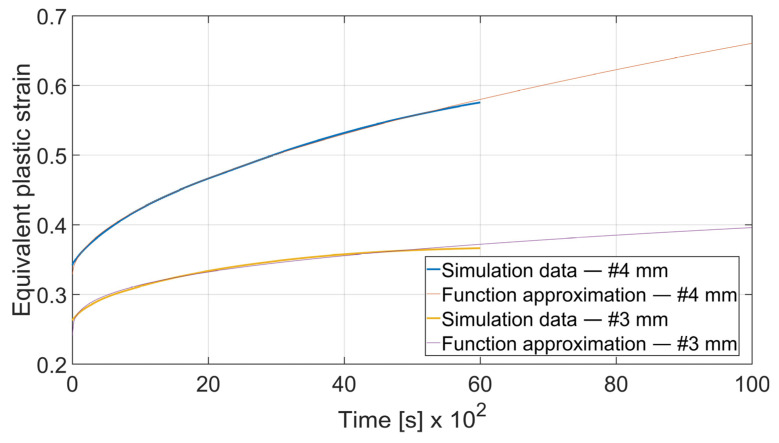
Equivalent plastic strain cumulation rate comparison for *α* = 115° and *θ* = 0°.

The fatigue resistance is predicted by evaluating the total equivalent strain cumulation in a given finite element during multiple loads. In each case, the element of maximal strain value at the time *t*_0_ = 0 s was chosen. Obtained results were approximated by the power function using the MathWorks (located in Natick, MA, USA) Matlab’s^®^ Curve Fitter App to show the strain cumulation beyond the simulation time. Function approximation confidence bounds were set to 95%. Strain cumulation curves are presented in Figure 17, Figure 18, Figure 19, Figure 20, Figure 21, Figure 22, Figure 23 and Figure 24. Regression functions were applied to shorten the computational time and obtain the optimal results accuracy in minimal computational time.

As shown in Figure 17 the strain development curve for the case of *x* = 4 mm, *α* = 90° differs significantly for *θ* = 0° and *θ* = 90°. Equivalent plastic strain in a given period of time can be expressed as (6) for bending along X direction (*θ* = 0°) and as (7) for bending along the Y direction (*θ* = 90°).

(6) $$\varepsilon_{cx}{}^{4;\;90{}^{\circ}}=2.149\cdot t^{0.3565}+0.4381$$

(7) $$\;\varepsilon_{cy}{}^{4;\;90{}^{\circ}}=1.227\cdot t^{0.4589}+0.4868$$

where *ε_cx_*—total equivalent plastic strain in case of bending with *θ* = 0°; *ε_cy_*—total equivalent plastic strain in case of bending with *θ* = 90°. Superscript denotes the material thickness and bending angle.

As it can be seen, bending with *θ* = 0° is a more favorable case as the ratio of strain cumulation is significantly lower. Thus, it can be stated that a specimen bent along the rolling direction will be more durable in terms of fatigue wear. A similar conclusion can be drawn by analyzing the case of bending angle *α* = 115°, as it is shown in Figure 18.

The regression functions for strain development for the case of *x* = 4 mm and *α* = 115° are presented in expressions (8)—*θ* = 0° and (9)—*θ* = 90°.

(8) $$\varepsilon_{cx}{}^{4;\;115{}^{\circ}}=2.687\cdot t^{0.5456}+0.3291$$

(9) $$\varepsilon_{cy}{}^{4;\;115{}^{\circ}}=2.044\cdot t^{0.6225}+0.3681$$

Specimen with thickness *x* = 3 mm and bending angle *α* = 90° showed the similar behavior as presented on Figure 19. However, the difference can be spotted in the case of *x* = 3 mm and *α* = 115°. In Figure 20 it can be clearly seen that after *t* = 4000 s total equivalent strain is higher in the specimen bent with *θ* = 0° despite the initial strain value being significantly lower. Strain development curves are shown on Equations (10)–(13).


(10) $$\varepsilon_{cx}{}^{3;\;90{}^{\circ}}=3.021\cdot t^{0.1711}+0.3274$$

(11) $$\varepsilon_{cy}{}^{3;\;90{}^{\circ}}=2.963\cdot t^{0.1406}+0.3595$$

(12) $$\varepsilon_{cx}{}^{3;\;115{}^{\circ}}=3.405\cdot t^{0.3292}+0.241$$

(13) $$\varepsilon_{cy}{}^{3;\;115{}^{\circ}}=3.754\cdot t^{0.236}+0.2672$$

To assess the influence of material thickness and bending angle on strain cumulation rate the given curves were compared. As shown in Figure 21 and Figure 22 increasing the bending angle increases the strain cumulation rate. In the case of *x* = 4 mm the equivalent plastic strain for a specimen bent on *α* = 115° surpasses the one for *α* = 90° after *t* = 3420 s and in the case of *x* = 3 mm after *t* = 9446 s.

Figure 23 and Figure 24 represent the strain cumulation comparison for different material thicknesses. In both cases, the cumulation rate is significantly higher for the thicker material.

It can be observed that some of the regression functions do not fit the simulation results optimally, especially in Figure 19, Figure 20 and Figure 22. Authors are aware of this fact, but the correlation coefficient for all of those functions is higher than 0.8. Furthermore, the fit error is more visible in the starting time period than in the end time. For the purpose of prognosing the fatigue life, it is more desirable to accurately predict the phenomena which occur in the late stage of the specimen life cycle.

## 4. Discussion

An analysis of stress maps in different specimens showed that stress distribution in bending operation is dependent on the material thickness, bending angle, and bending line orientation to the sheet rolling direction. In a qualitative manner, it was proven that stress distribution varies for *θ* = 0° and *θ* = 90° due to the sheet anisotropy in all analyzed cases. Additionally, in a quantitative manner, changing the θ angle resulted in the maximal stress value registered in the process. In the case of *α* = 90° maximal stress value was higher by 7.82% and 7.81% accordingly for *x* = 4 mm and *x* = 3 mm. It can be also observed that the stress growth is even higher in the case of *α* = 115° and it equals 8.58% for *x* = 4 mm and 8.49% for *x* = 3 mm.

Similar conclusions can be drawn by analyzing the equivalent plastic strain distribution maps and maximal values. Although strain distribution is not much different in a qualitative manner it can be observed a significant change in maximum value when changing the *θ* angle. Similarly, like in the stress case, the change is more noticeable in the case of bending with *α* = 115°.

Stress and strain analysis showed that those values and their distribution are dependent on material thickness, bending angle, and the angle between the rolling direction and bending line, which comply with the works mentioned in the introduction [7,8,9,10,11,12]. It is also believed that those changes influence the springback phenomenon.

In this work, it was confirmed that the springback coefficient—*K* depends on the *θ* angle and it has the lowest value when *θ* = 0° like in works [7,8,9,10]. Additionally, the material thickness is a considerable factor when predicting springback as in [11,12]. Reducing the thickness results in increasing the *K* value.

Model validation in both qualitative and quantitative ways showed its value for predicting the phenomena occurring in the bending zone. Based on this, a new approach to predicting total equivalent strain in the bending zone during the multiple bending test was proposed.

It can be observed that specimens bent along the rolling direction (*θ* = 0°) showed significantly lower strain cumulation rate. It is believed this influences the fatigue life of samples. A lower cumulation rate is believed to be a more favorable case in terms of fatigue life. Thus, it is suggested to align the bending line with the sheet rolling direction whenever it is possible to obtain the best expected fatigue resistance.

As it can be observed analyzed load conditions resulted in a higher strain cumulation rate in specimens bent with *α* = 115° than in the ones bent with *α* = 90°. In this case, authors believe this is specific to given load conditions and should be further investigated to determine strain cumulation rates dependent on them.

In given conditions, it was proven that decreasing the material thickness positively affected the rate of strain cumulation. It could be assumed that in a given case reducing the thickness could be positive in many ways. Especially in terms of expected fatigue lifetime. Of course, it is not possible to constantly decrease thickness due to the design requirements and strength properties of specific parts.

## 5. Conclusions

In this work, the analysis of the air-bending process of the anisotropic DC01 steel was performed. By usage of Barlat’s anisotropic plasticity model, the stress and strain distribution maps in specimens were assessed in various bending conditions. This paper also shows the analysis of springback coefficient analysis and strain cumulation in the bending zone during iterative loads. The main objective of this work was to develop an ability to forecast the fatigue lifetime of cold-formed angles in terms of selected process parameters. The conducted study allowed for developing the regression functions of strain cumulation in the bending zone, which could be helpful in the prediction of specimen fatigue life length. The main objective was fulfilled and set the ground for new research directions. Further studies will be concerned with strain measurements and the search for universal guidelines for selecting sheet metal bending process parameters in terms of providing the longest possible service life and ensuring extended service intervals.

## Figures and Tables

**Table 1:** Chemical composition of DIN EN 10130 DC01 steel.

C Max. [%]	P Max. [%]	S Max. [%]	Mn Max. [%]	Ti Max. [%]
0.120	0.045	0.045	0.600	-

**Table 2:** Material parameters used in the simulation.

*ρ* [kg/m^3^]	*E* [GPa]	*ν* [-]	*k* [-]	*ε*_0_ [-]	*n* [-]	*m* [-]	*a* [-]	*b* [-]	*c* [-]	*f* [-]	*g* [-]	*h* [-]
8000	210	0.30	573	0.01	0.30	2	0.70	0.88	1.20	1	1	1

where *ρ*—mass density, *E*—Young’s modulus, *ν*—Poisson’s ratio, *k*—strength coefficient, *ε*_0_—strain corresponding to the initial yield, *n*—hardening exponent for yield strength, *m*—flow potential exponent, *a*, *b*, *c*, *f*, *g*, *h*—Barlat’s model anisotropy coefficients.

**Table 3:** Comparison of specimen’s bending zone width in simulation and experiment.

Bending Angle— *α* [°]	Angle between Bending Line and Rolling Direction—*θ* [°]	Specimen Width from Simulation—*w_s_* [mm]	Specimen Width from Measurement—*w_m_* [mm]	$\mathrm{Width}\;\mathrm{Difference}—\Delta w\;(\left|w_{s}-w_{m}\right|)\;[\mathrm{mm}]$	Error—e $\left(\frac{\Delta w}{w_{m}}\cdot 100\%\right)\;[\%]$
90	0	20.728	20.600	0.128	0.62
	90	20.798	20.575	0.223	1.08
115	0	20.665	20.475	0.190	0.93
	90	20.727	20.366	0.361	1.77

**Table 4:** Comparison of springback coefficient *K* in simulation and experiment.

Bending Angle— *α* [°]	Angle between Bending Line and Rolling Direction—*θ* [°]	Springback Coefficient from Simulation—*K_s_* [-]	Springback Coefficient from Measurement—*K_m_* [-]	$\mathrm{Springback}\;\mathrm{Coefficient}\;\mathrm{Difference}—\Delta K\;(\left|K_{s}-K_{m}\right|)$ [-]	Error—e_K_ $\left(\frac{\Delta K}{K_{m}}\cdot 100\%\right)\;[\%]$
90	0	1.0180	1.0460	0.028	2.68
	90	1.0210	1.0390	0.018	1.73
115	0	1.0129	1.0490	0.036	3.44
	90	1.0142	1.0510	0.037	3.52

**Table 5:** HMH equivalent stress change in bending with different *θ* angle (*x* = 4 mm).

Bending Angle—*α* [°]	Angle between Bending Line and Rolling Direction—*θ* [°]	Maximal Stress Value [MPa]	Stress Value Change [%]
90	0	532.2	7.82
	90	573.8	
115	0	486.1	8.58
	90	527.8	

**Table 6:** HMH equivalent stress change in bending with different *θ* angle (*x* = 3 mm).

Bending Angle—*α* [°]	Angle between Bending Line and Rolling Direction—*θ* [°]	Maximal Stress Value [MPa]	Stress Value Change [%]
90	0	500.9	7.81
	90	540	
115	0	463.5	8.49
	90	502.8	

**Table 7:** Equivalent plastic strain change in bending with different *θ* angle (*x* = 4 mm).

Bending Angle—*α* [°]	Angle between Bending Line and Rolling Direction—*θ* [°]	Maximal Strain Value [mm/mm]	Strain Value Change [%]
90	0	0.4491	8.97
	90	0.4894	
115	0	0.3270	10.86
	90	0.3625	

**Table 8:** Equivalent plastic strain change in bending with different *θ* angle (*x* = 3 mm).

Bending Angle—*α* [°]	Angle between Bending Line and Rolling Direction—*θ* [°]	Maximal Strain Value [mm/mm]	Strain Value Change [%]
90	0	0.3428	8.61
	90	0.3723	
115	0	0.2627	10.47
	90	0.2902	

**Table 9:** Results of the springback coefficient calculations.

Bending Angle—*α* [°]	Material Thickness—*x* [mm]	Angle between Bending Line and Rolling Direction—*θ* [°]	Springback Coefficient— *K* [-]
90	3	0	1.0180
		90	1.0210
	4	0	1.0140
		90	1.0150
115	3	0	1.0129
		90	1.0142
	4	0	1.0093
		90	1.0099

## Data Availability

Data are available from authors upon request.
